# Clinical course and management of idiopathic pulmonary fibrosis

**DOI:** 10.1186/s40248-019-0197-0

**Published:** 2019-12-02

**Authors:** Caitlin Quinn, Amy Wisse, Stephenie T. Manns

**Affiliations:** 1 Emory Critical Care Center, 1364 Clifton Road, NE, Atlanta, GA 30322 USA; 2 Medical University of South Carolina (MUSC), 25 Courtenay Drive, MSC 114, Charleston, SC 29425 USA; 3 Pulmonary and Critical Care Medicine, Wake Med Brier Creek Healthplex, Brier Creek, 8001 TW Alexander Drive, Suite 218, Raleigh, NC 27617 USA

**Keywords:** Dyspnea, Fibrosis, Idiopathic pulmonary fibrosis, Interstitial lung disease

## Abstract

Idiopathic pulmonary fibrosis (IPF) is a progressive, fatal interstitial lung disease (ILD) with an unpredictable clinical course. Although IPF is rare, healthcare professionals should consider IPF as a potential cause of unexplained chronic dyspnea and/or cough in middle-aged/elderly patients and refer patients to a pulmonologist for evaluation. Making a diagnosis of IPF requires specialist expertise. Multidisciplinary discussion, involving at minimum a pulmonologist and a radiologist with expertise in the differential diagnosis of ILDs, is required to ensure the most accurate diagnosis. Prompt diagnosis of IPF is important to enable patients to receive appropriate care from an early stage. Optimal management of IPF involves the use of antifibrotic drugs, as well as the provision of supportive care to alleviate symptoms and preserve patients’ quality of life. Antifibrotic drugs have been shown to slow lung function decline seen in patients with IPF. Patients’ symptoms and functional capacity can be improved through participation in pulmonary rehabilitation programs and the use of supplemental oxygen. Patient education is essential to help patients understand and manage their disease. The identification and management of comorbidities, such as obstructive sleep apnea, pulmonary hypertension, and emphysema, is also an important element of the overall care of patients with IPF. Patients with IPF should be evaluated for lung transplantation at an early stage to maximize their chances of meeting eligibility criteria. In this review, we describe the clinical course and impact of IPF and best practice in its management, highlighting the importance of taking a patient-centered approach.

## Background

IPF is an interstitial lung disease (ILD) of unknown cause characterized by progressive fibrosis of the lungs with a radiologic or histopathologic pattern known as usual interstitial pneumonia (UIP) [1]. IPF is a rare disease, but its reported incidence appears to be increasing as it becomes more widely recognized [2]. IPF is more common in current/former smokers, in men, and usually presents in patients in their sixties [3]. Progression of IPF is characterized by decline in lung function, worsening symptoms of dyspnea and cough, and deterioration in quality of life. As the disease progresses, patients’ ability to perform everyday activities becomes increasingly impaired and the social and emotional impact of the disease takes its toll [4–6]. Given this, care given to patients with IPF should be multi-faceted and attempt to improve the quality of life of the patient while slowing decline in lung function. In this review, we will describe the clinical course and impact of IPF and best practice in its management, highlighting the importance of taking a patient-centered approach to care.

### Natural history of IPF

**Fig. 1:**
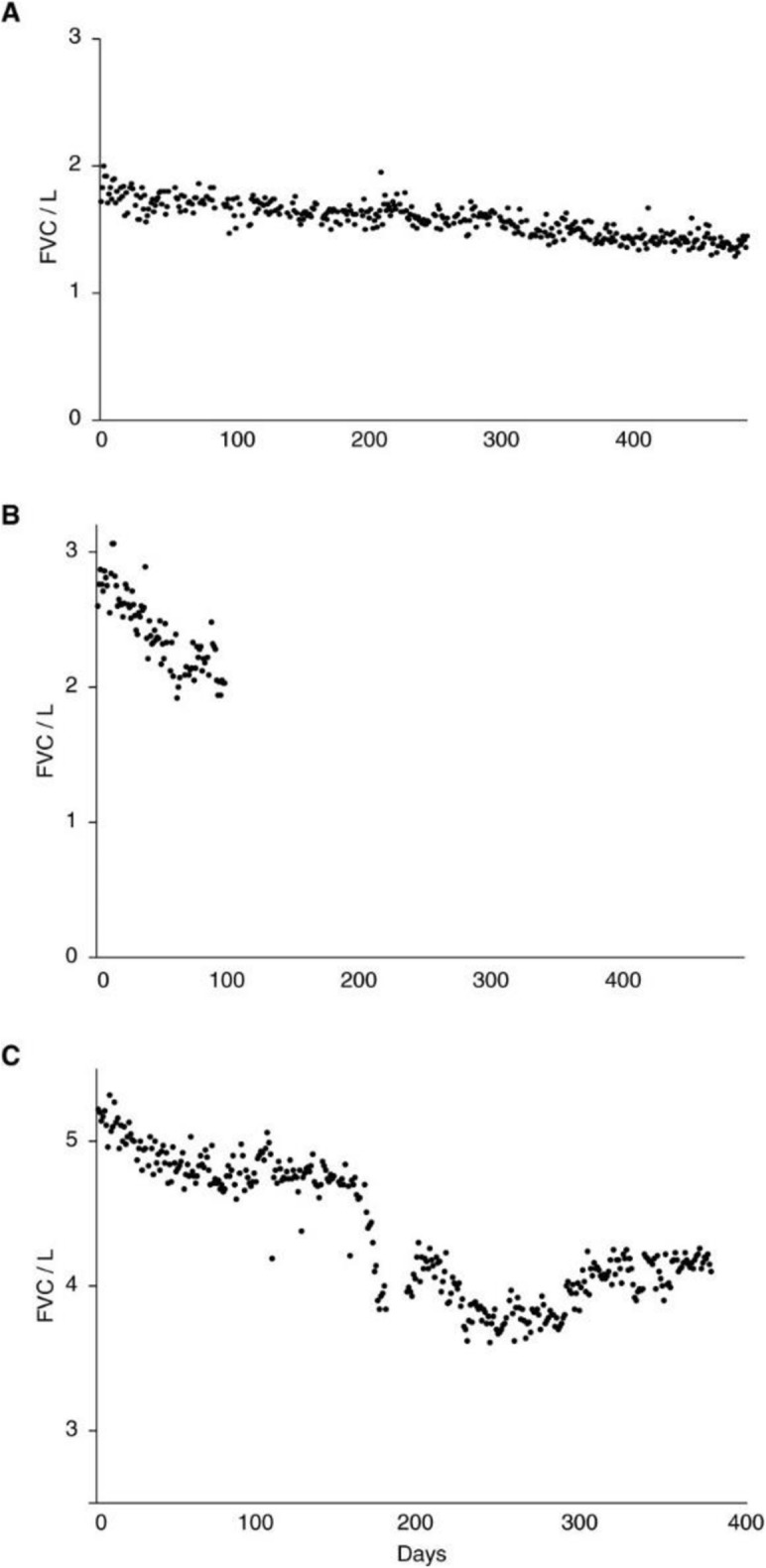
Examples of disease course in patients with IPF. Daily FVC measurements for subjects with (**a**) inexorably progressive disease, (**b**) rapidly progressive disease, and (**c**) an acute exacerbation. Each point represents a single FVC measurement. From: Russell et al., 2016 [8]. Reprinted with permission of the American Thoracic Society. Copyright© 2018 American Thoracic Society. Russell AM, et al./2016/Daily home spirometry: an effective tool for detecting progression in idiopathic pulmonary fibrosis/Am J Respir Crit Care Med/194/989–997. The American Journal of Respiratory and Critical Care Medicine is an official journal of the American Thoracic Society

IPF has a variable clinical course [7]. Some patients progress relatively slowly; others have a rapid decline in lung function leading to death; and others suffer a stepwise loss of lung function with periods of relative stability (Fig. 1). Acute deteriorations in respiratory function in patients with IPF, known as acute exacerbations, usually result in hospitalization and are associated with very high mortality [9]. The median survival of patients with IPF who experience an acute exacerbation is approximately 3 to 4 months [9]. Although the course of IPF is variable, its overall prognosis is poor, with a median post-diagnosis survival in patients not receiving antifibrotic therapy or lung transplant of only 3–4 years [2, 10].

**Fig. 2:**
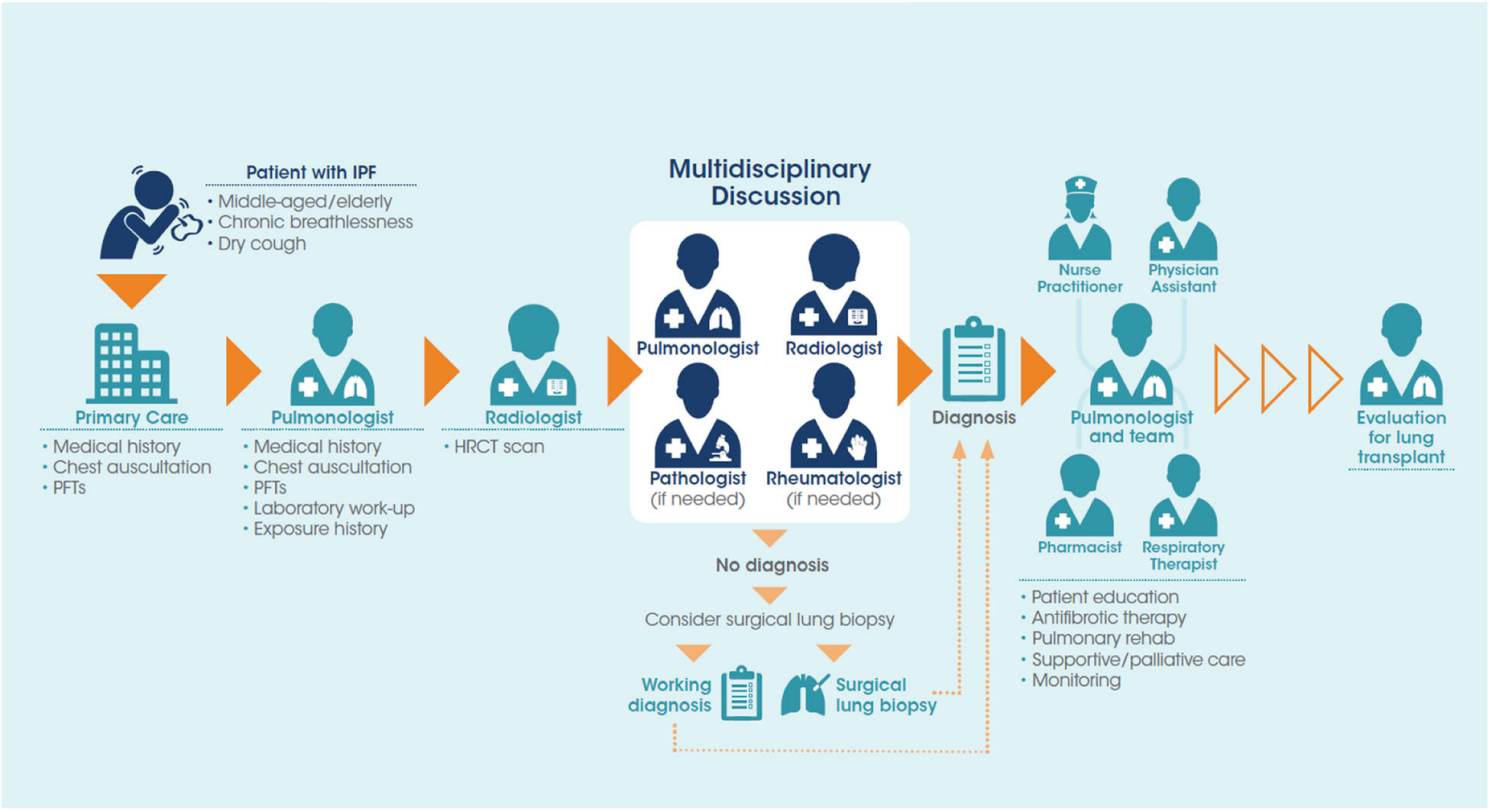
The patient journey in idiopathic pulmonary fibrosis

Patients are often delayed in receiving a diagnosis of IPF, as its non-specific symptoms are easily mistaken for those of more common diseases such as chronic obstructive pulmonary disease (COPD), gastroesophageal reflux disease (GERD), or heart disease [11, 12]. It is important that healthcare professionals consider IPF as a potential diagnosis in middle-aged/elderly patients who present with unexplained chronic dyspnea and/or cough. Bilateral inspiratory crackles that sound like “Velcro” being torn apart on chest auscultation are an early sign of IPF and should increase a clinician’s index of suspicion for this disease [13]. Patients with a suspected ILD should be promptly referred to a pulmonologist with expertise in the diagnosis of ILDs. Making a diagnosis of IPF is challenging and requires specialist expertise to exclude other conditions that have a similar clinical and radiological presentation, such as chronic hypersensitivity pneumonitis or ILDs related to autoimmune diseases [14]. Multidisciplinary discussion, involving a pulmonologist, radiologist and, when appropriate, a pathologist and/or rheumatologist, is recommended to enable the most accurate diagnosis to be made [1, 15] (Fig. 2).

Several large studies have identified predictors of mortality in patients with IPF, such as decline in forced vital capacity (FVC), diffusion capacity for carbon monoxide (DLco), or 6-min walk distance [16–18]. The GAP index and staging system is a simple categorization method, developed based on three cohorts of patients in real-world settings, that can be used to predict mortality in patients with IPF based on gender, age, FVC % predicted, and DLco % predicted [19]. However, none of the tools that are currently available enable a robust prediction of the course of disease to be made at diagnosis. In particular, preservation of FVC at any given time point is no guarantee that FVC will continue to remain stable and acute exacerbations can occur even in patients with well-preserved lung function [20]. This creates challenges for healthcare professionals in counseling patients about what they should expect in the months or years following a diagnosis of IPF. Regular follow-up visits with frequent monitoring of pulmonary function and 6-min walk tests are important to monitor how a patient’s disease is progressing and to enable timely discussions to be held about potential changes to their care, including discussion of palliative care when needed.

### Pharmacological treatments

**Fig. 3:**
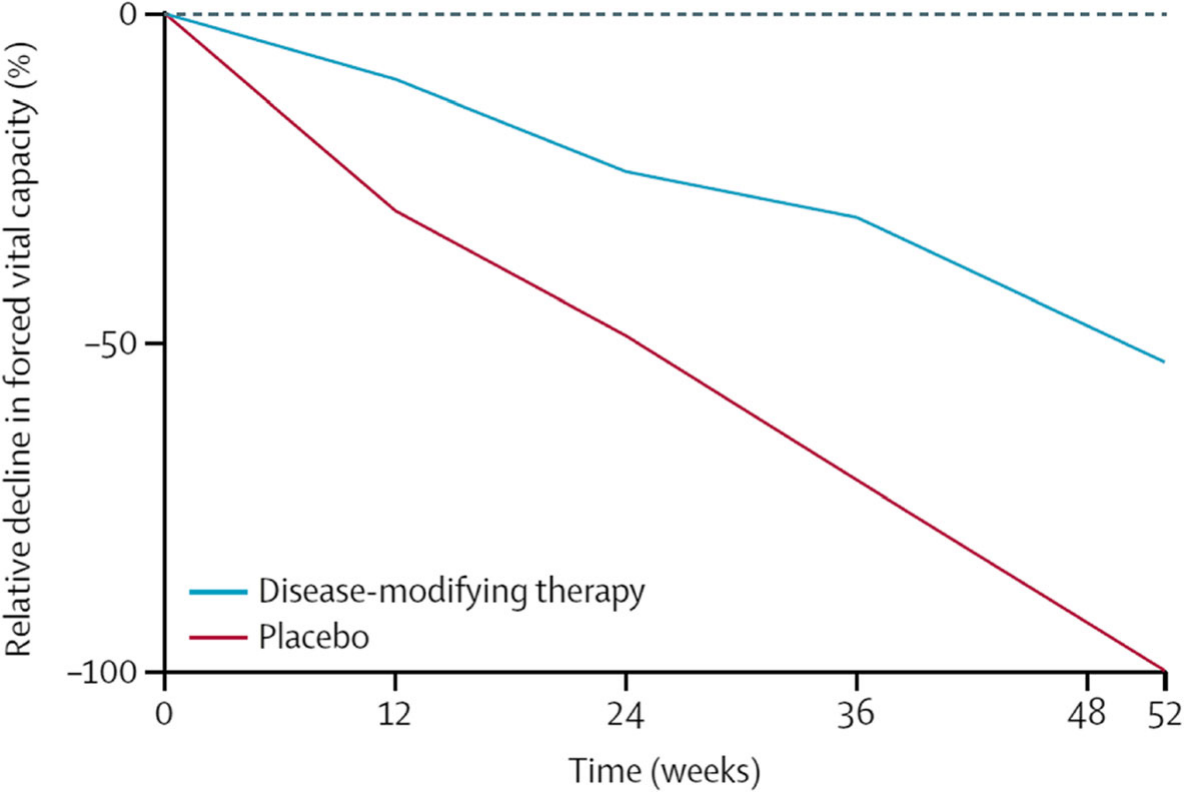
Effect of antifibrotic therapies on lung function decline [24]. Reprinted from The Lancet, Vol. 389, Richeldi L, et al., Idiopathic pulmonary fibrosis, Page No. 1941–1952., Copyright (2017), with permission from Elsevier

In most countries, two drugs are approved for the treatment of IPF: nintedanib and pirfenidone. Both of these drugs received conditional recommendations for use in the latest international treatment guideline for IPF, indicating that they are appropriate choices for a majority of patients, while acknowledging that patients’ preferences should be taken into account when making therapeutic decisions [21]. Studies conducted in cellular systems and animal models of lung fibrosis suggest that nintedanib and pirfenidone inhibit processes that are fundamental to the progression of fibrosis such as the proliferation, migration and differentiation of fibroblasts and the deposition of extracellular matrix components such as collagen in the lungs [22, 23]. Large clinical trials have shown that in patients with IPF with mild or moderate impairment in lung function (FVC > 50% predicted), nintedanib and pirfenidone reduce the rate of decline in FVC by approximately 50% over 1 year of treatment (Fig. 3) [25, 26]. Importantly, the effect of these drugs on reducing decline in FVC has been shown to be consistent across the spectrum of baseline FVC studied and across subgroups by age, race, gender and concomitant medication use [20, 27–30]. Long-term data from the open-label extension trials of the randomized placebo-controlled trials of nintedanib [30] and pirfenidone [31] suggest that the reductions in FVC decline persist for several years, with no new safety signals identified .

In addition to reducing the rate of FVC decline, there is some evidence that nintedanib and pirfenidone may reduce the risk of severe acute deteriorations in lung function [32, 33] and improve life expectancy [34, 35]. However, neither drug provides significant relief from the symptoms of IPF, nor an improvement in patients’ quality of life. It is important that healthcare professionals manage patients’ expectations of treatment by explaining to them that the aim of drug therapy is to slow the progression of their disease and that it is unlikely that they will see an improvement in their lung function or symptoms.

The side-effect profiles of nintedanib and pirfenidone are characterized predominantly by gastrointestinal events such as diarrhea, nausea and vomiting [36–38]. By advising patients on the adverse events that may be associated with antifibrotic treatment and the most appropriate ways to manage them, healthcare professionals play a key role in helping patients with IPF remain on therapy. In most patients, the gastrointestinal side-effects associated with these drugs can be managed through dose adjustment, treatment interruption, and measures to manage symptoms, including adequate hydration and the use of loperamide or antiemetics. Importantly, the dose adjustments used to manage the side effects of nintedanib and pirfenidone have been shown not to reduce the efficacy of these medications in reducing lung function decline [39, 40].

**Fig. 4:**
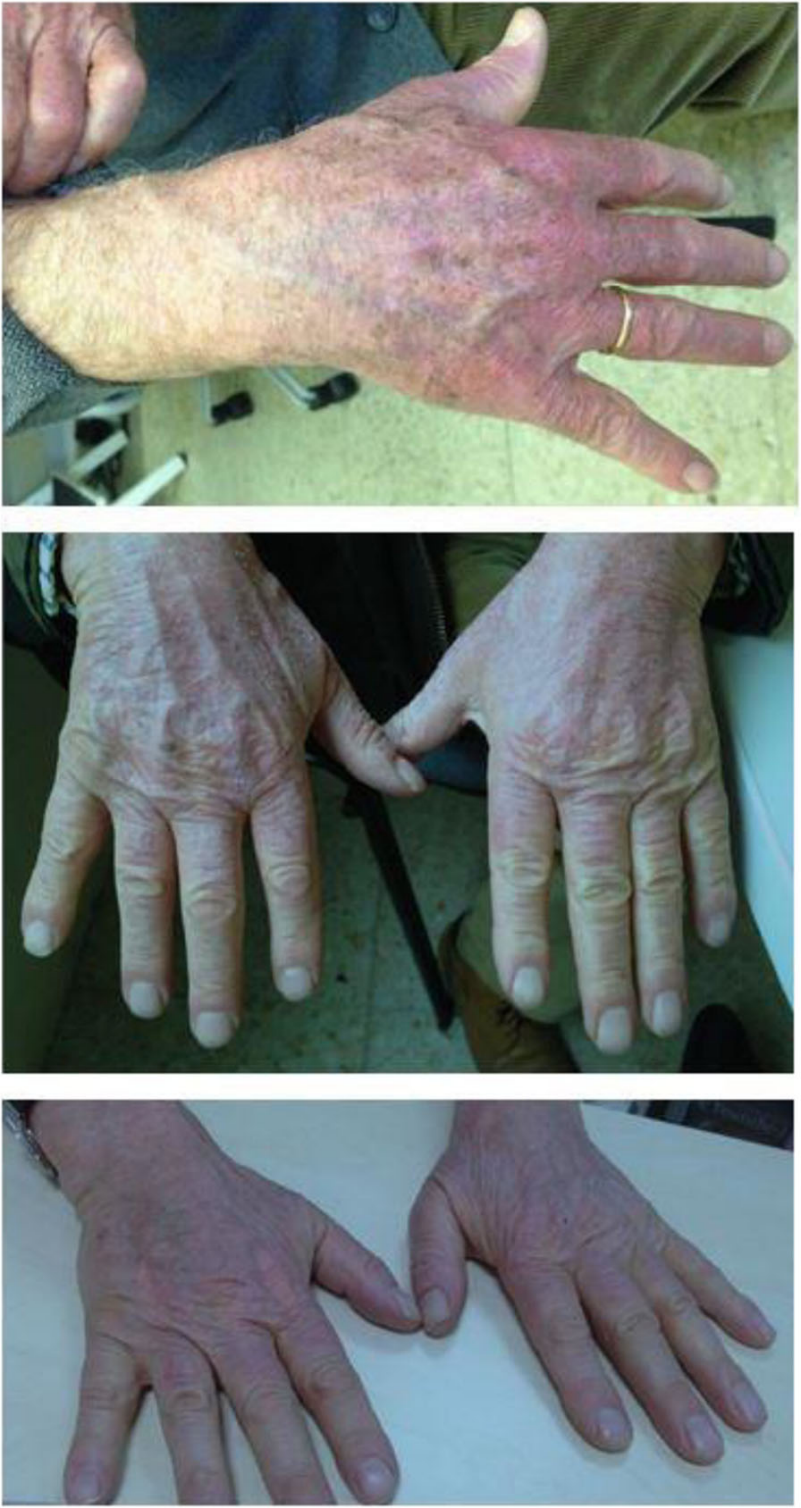
Photosensitivity in pirfenidone-treated patients [41]. Reprinted by permission from RightsLink®: Springer, Advances in Therapy, 31(4):375–91, Pirfenidone in idiopathic pulmonary fibrosis: expert panel discussion on the management of drug-related adverse events, Costabel U, et al., Copyright® 2014

Elevations in liver enzymes occur in a small proportion of patients treated with antifibrotic therapy. It is recommended that liver enzymes be monitored during the first 3 months of nintedanib treatment, and periodically thereafter or as clinically indicated. Liver enzymes should be monitored monthly for the first 6 months of pirfenidone treatment, and every 3 months thereafter. In addition, it is important that liver function is measured promptly in patients who experience symptoms that may be a sign of liver injury, including fatigue, anorexia, right upper abdominal discomfort, dark urine, or jaundice. Treatment with pirfenidone may be associated with photosensitivity and rash (Fig. 4) so it is recommended that patients avoid exposure to sunlight and sunlamps, and wear sunscreen and protective clothing.

Arterial thromboembolic events have been reported in patients taking nintedanib, and it is recommended that caution be used when treating patients at higher cardiovascular risk, including known coronary artery disease. In the Phase III INPULSIS trials, a higher proportion of patients in the nintedanib than the placebo group had myocardial infarction (2.7% versus 1.2%), whereas a lower proportion of patients in the nintedanib than the placebo group had other ischemic heart disease (1.7% versus 3.1%) [42]. Reassuringly, the incidence rate of myocardial infarction reported in post-marketing surveillance in the US in the year following the launch of nintedanib as a treatment for IPF (10 per 1000 patient–years) was lower than that observed in patients treated with nintedanib in the INPULSIS trials (17 per 1000 patient–years) [43].

As an inhibitor of the vascular endothelial growth factor receptor [22], nintedanib is associated with an increased risk of bleeding. Thus patients at known risk of bleeding, including those treated with full-dose anticoagulants or high-dose antiplatelet therapy, were excluded from clinical trials of nintedanib. In the Phase III INPULSIS trials, bleeding events were reported in 10 and 8% of patients with IPF treated with nintedanib and placebo, respectively, with nose-bleed being the most commonly observed type of bleeding event [42]. In US post-marketing surveillance data, the overall incidence of bleeding, and the most common types of bleeding events, were very similar to observations in nintedanib-treated patients in the INPULSIS trials [43].

Other than nintedanib and pirfenidone, the medications that are commonly used to treat IPF are not supported by a robust evidence base. A 24-week randomised trial of nintedanib plus sildenafil versus nintedanib alone in patients with IPF and severe impairment in gas exchange found that the combination provided no significant benefit on quality of life compared with nintedanib alone, but was associated with a numerical benefit on FVC decline [44]. The use of anti-acid medications in patients with IPF and asymptomatic GERD was given a conditional recommendation in the latest international treatment guidelines [21], but remains controversial given the lack of randomized controlled trials to support their efficacy in IPF and concerns over potential safety issues [29, 45–47]. Recently, a small randomised trial conducted in IPF patients with abnormal acid levels confirmed by 24-h pH testing found that there was no significant difference in FVC decline over 48 weeks in patients who did (*n* = 27) and did not (*n* = 20) undergo laparoscopic anti-reflux surgery [48]. Triple therapy with prednisone, azathioprine, and N-acetylcysteine (NAc), once regarded as standard of care for IPF, was shown in a large placebo-controlled trial to be harmful and should be avoided [49]. There is no evidence to support the use of immunosuppressants as a chronic therapy for patients with IPF, although international guidelines provide a weak recommendation for the use of corticosteroids in patients with an acute exacerbation based on very low-quality evidence [50]. Anticoagulants appear to be harmful to patients with IPF and should not be used in its treatment [21, 51, 52].

The antioxidant NAc is a popular therapy in patients with IPF and has a good safety and tolerability profile, but the largest trial of NAc in patients with IPF showed no efficacy of NAc compared to placebo in reducing FVC decline over 60 weeks of treatment [53]. Further, there is some evidence, albeit from a single 24-week study, that the addition of NAc to pirfenidone therapy may reduce the effectiveness of pirfenidone in reducing lung function decline, possibly by reducing its bioavailability [54].

### Supportive care

**Fig. 5:**
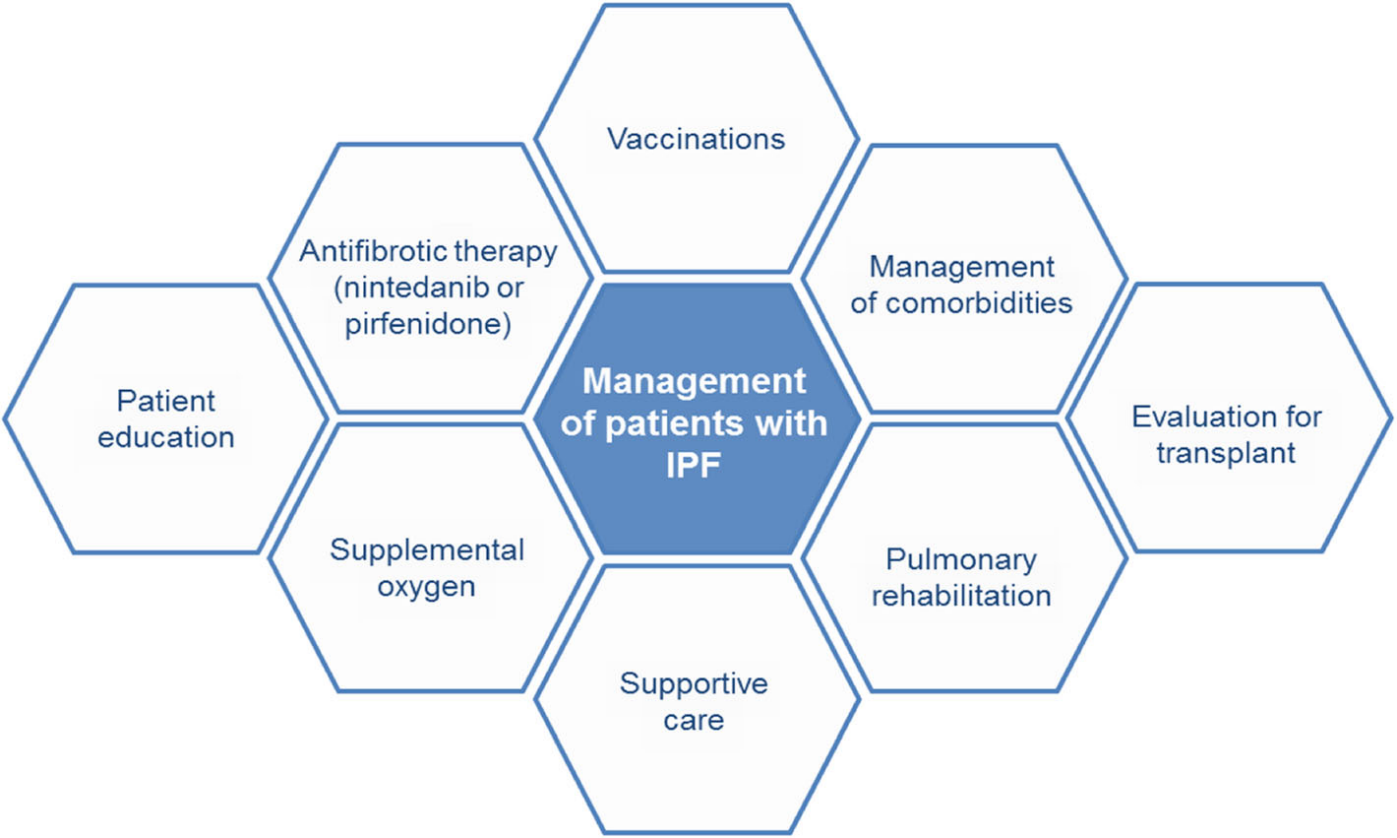
A holistic approach to the management of patients with IPF

Patients with IPF benefit from a holistic approach to care that goes beyond drug therapy and also encompasses patient education, symptom relief, and the management of comorbidities (Fig. 5). Pulmonary rehabilitation has been shown to provide short-term benefits in regards to dyspnea, functional capacity and quality of life [55, 56] and is recommended in international treatment guidelines [21]. The cornerstone of pulmonary rehabilitation is exercise training, which may involve aerobic exercise (e.g. cycling or walking), resistance training, flexibility training and advice on breathing techniques. Supplemental oxygen is recommended for patients with IPF and significant resting hypoxemia, commonly defined as a resting SpO_2_ of < 88%, and is frequently used by patients with advanced disease, but more research is needed to establish its impact on patients [57]. There is evidence that supplemental oxygen improves exercise capacity and quality of life in patients with ILD, but its effects on exertional dyspnea are inconsistent [57, 58].

The use of opioids to provide symptomatic relief in patients with IPF remains controversial, with few data available to guide therapeutic decision-making. When administered systemically, opioids have been associated with an improvement in dyspnea and exercise capacity in patients with IPF; however, side effects like constipation should be monitored [59]. Data from large randomized placebo-controlled trials are needed to establish the risk:benefit of opioid use in patients with IPF. The cough associated with IPF poses major challenges to patients and clinicians as it is often distressing for patients and is refractory to typical anti-tussive treatments [60]. Vaccines against pneumonia, influenza, and other respiratory infections should be offered to all patients with IPF, as such infections can have a serious impact on patients with impaired lung function and may result in an acute exacerbation. Patients should also be educated about infection prevention practices, such as hand hygiene and avoiding contact with people known to be unwell.

The identification and management of comorbidities such as GERD, pulmonary hypertension, cardiovascular disease, COPD/emphysema, and sleep apnea is an important part of the overall care of patients with IPF and affect prognosis [61]. It is important that healthcare professionals take a proactive approach to diagnosing and managing comorbidities in patients with IPF, as this can bring substantial benefits; for example, management of cough due to a comorbidity, or helping a patient to improve their sleep, might substantially improve a patient’s quality of life. Polypharmacy should be borne in mind as many patients with IPF are elderly and are taking several medications, which may reduce adherence to treatment and increase the risk of adverse events [62]. Depression and anxiety are often overlooked in patients with IPF yet can have a profound impact on quality of life [5]. Issues around patient frailty should also be considered.

Patient education and support are essential to helping patients deal with the consequences of living with IPF. Patients and their families need information at the time of diagnosis, but there may be value in pacing the delivery of some information as the disease progresses [63, 64]. Healthcare professionals need to bear in mind that most patients will have read information about IPF on the Internet that has scared them and that may well be inaccurate or out of date [65]. Qualitative research based on semi-structured interviews with patients with IPF has described the value of support groups in helping patients to develop problem-focused coping strategies on how to live with their condition [66]. Local or national patient support groups such as the Pulmonary Fibrosis Foundation (www.pulmonaryfibrosis.org) can be of great help in providing educational resources, advice and emotional support to patients and their caretakers. In addition, patients taking an antifibrotic drug have access to 24/7 support lines staffed by trained nursing professionals, which provide general information on IPF as well as information on drug therapies (https://www.ofev.com/support/open-doors; https://www.esbriethcp.com/ipf-patient-assistance/patient-education-inspiration.html).

All patients with IPF should have access to palliative care, which should include a careful assessment of the patient’s priorities and respect the preferences of the patient and their family [67]. An early and multidisciplinary approach to the provision of palliative care in the outpatient setting may help reduce hospitalizations and make it more likely that patients who wish to do so can die at home rather than in hospital [68].

### Lung transplantation

Lung transplantation offers a survival advantage to select patients with IPF and should be considered as an option for patients who meet eligibility criteria. Recent data from the registry run by the International Society for Heart and Lung Transplantation suggest that median post-transplant survival in patients with IPF is approximately 5 years [69]. Survival following lung transplant is slightly worse in patients with restrictive lung diseases than in patients with other indications for transplant [70]. International guidelines recommend that patients with IPF be evaluated for lung transplant at an early stage, due to the progressive and unpredictable nature of the disease [50, 71]. Early referral also enables initiation of patient education and allows time to address potential barriers to transplantation, such as obesity, nutritional status, deconditioning, and poor control of other medical conditions [71, 72].

## Conclusions

Management of IPF should be multi-faceted, multi-disciplinary and patient-centric. Antifibrotic therapies reduce the rate at which IPF progresses but do not alleviate the symptoms of the disease. Pulmonary rehabilitation and the use of supplemental oxygen can help to relieve dyspnea and improve patients’ quality of life. Measures to prevent infections are important to reduce the risk of acute exacerbations of the disease, which are associated with high morbidity and mortality. Healthcare professionals play a key role in helping patients, families, and caregivers live with the consequences of IPF from diagnosis until end of life.

## Data Availability

Not applicable.

## Abbreviations

COPD: Chronic obstructive pulmonary disease
DLco: Diffusion capacity for carbon monoxide
FVC: Forced vital capacity
GERD: Gastroesophageal reflux disease
ILD: Interstitial lung disease
IPF: Idiopathic pulmonary fibrosis
NAc: N-acetylcysteine
UIP: Usual interstitial pneumonia
